# Lipids, lipid-lowering drugs and lateral epicondylitis of the humerus: a drug-targeted Mendelian randomization study

**DOI:** 10.3389/fgene.2024.1437712

**Published:** 2024-09-02

**Authors:** Meng-Meng Liu, Xiang Chen, Xiao-Hang Bao, Bao-Hua Huang

**Affiliations:** ^1^ School of Physical Education And Health, Guangxi Medical University, Nanning, China; ^2^ Department of Bone and Joint Surgery, The First Affiliated Hospital of Guangxi Medical University, Nanning, China; ^3^ Department of Spinal Surgery, The First Affiliated Hospital of Guangxi University of Chinese Medicine, Nanning, China

**Keywords:** lipids, lipid-lowering drugs, lateral epicondylitis, Mendelian randomization, drug target

## Abstract

**Background:**

Clinical observations indicate that blood lipids may be risk factors for lateral epicondylitis (LE) of the humerus, and lipid-lowering drugs are also used for the prevention and treatment of tendon diseases, but these lack high-quality clinical trial evidence and remain inconclusive. Mendelian randomization (MR) analyses can overcome biases in traditional observational studies and offer more accurate inference of causal relationships. Therefore, we employed this approach to investigate whether blood lipids are risk factors for LE and if lipid-lowering drugs can prevent it.

**Methods:**

Genetic variations associated with lipid traits, including low-density lipoprotein cholesterol (LDL-C), triglycerides (TG), and total cholesterol (TC), were obtained from the UK Biobank and the Global Lipids Genetics Consortium (GLGC). Data on genetic variation in LE were sourced from FinnGen, including 24,061 patients and 275,212 controls. Subsequently, MR analyses were conducted to assess the potential correlation between lipid traits and LE. Additionally, drug-target Mendelian randomization analyses were performed on 10 drug targets relevant to LE. For those drug targets that yielded significant results, further analysis was conducted using colocalization techniques.

**Results:**

No correlation was found between three blood lipid traits and LE. Lipoprotein lipase (LPL) enhancement is significantly associated with a decreased risk of LE (OR = 0.76, [95% CI, 0.65–0.90], *p* = 0.001). The expression of LPL in the blood is associated with LE and shares a single causal variant (12.07%), greatly exceeding the probability of different causal variations (1.93%), with a colocalization probability of 86.2%.

**Conclusion:**

The three lipid traits are not risk factors for lateral epicondylitis. LPL is a potential drug target for the prevention and treatment of LE.

## 1 Introduction

Lateral epicondylitis (LE) of the humerus, also known as “tennis elbow,” is primarily linked to overuse of the elbow, particularly affecting the extensor carpi radialis brevis muscle (Bisset and Vicenzino, 2015; Herquelot et al., 2013; Sayampanathan et al., 2020). Among adults, the incidence rate ranges from 1%–3%, while tennis players exhibit a significantly higher incidence, reaching 40%–50% (Sanders et al., 2015; Cutts et al., 2020; Stegink-Jansen et al., 2021). Surgical intervention is reserved for patients who resist conventional treatments (Knutsen et al., 2015; Xu et al., 2023). Typically, patients undergo conservative treatment, which includes rest, oral nonsteroidal anti-inflammatory drugs (NSAIDs), and localized corticosteroid injections. Prolonged NSAID usage may result in gastrointestinal complications (Wolf, 2023), whereas extended corticosteroid injections can lead to local skin and muscle atrophy, and pigmentation changes, among other concerns (Titchener et al., 2015; Branson et al., 2017; Kheiran et al., 2021; Kim et al., 2021). Therefore, there is an urgent need to develop new drugs to treat this disease.

Previous studies have shown a correlation between tendon diseases and hyperlipidemia (Tilley et al., 2015; Lee et al., 2019). The mechanism may involve lipid effects on tendon homeostasis, tendon remodeling, and inflammation activation (Lee et al., 2019; Steplewski et al., 2019; Chen et al., 2023). However, findings regarding the association between LE and blood lipids vary, while a meta-analysis indicates that high cholesterol levels are risk factors for LE (Chen et al., 2023), other studies suggest no clear association (Sayampanathan et al., 2020). Currently, research on the relationship between hyperlipidemia and LE remains limited. Observational studies, which dominate the available research, are prone to limitations and confounding factors, underscoring the incompleteness of our understanding of this relationship and the need for further exploration.

Statins are commonly used for cardiovascular diseases. Recent literature also suggests their potential in preventing tendon disorders (Yang and Qu, 2018; Lee et al., 2023). A systematic review and a cohort study with over 10 years of follow-up have shown that statins can reduce the risk of tendon disorders in patients with hyperlipidemia (Lin et al., 2015; Teichtahl et al., 2016). However, the gold standard for determining drug efficacy is randomized controlled trials (RCT), and currently, no relevant RCT studies exist. Therefore, further exploration of the relationship between lipid-lowering drugs and LE is necessary.

Mendelian randomization is a method that utilizes genetic variations as instrumental variables (IVs). The distribution of these variations in human populations is random, akin to the ideal conditions of an RCT. This randomness helps in investigating the causal relationship between exposure and disease, reducing confounding biases inherent in traditional observational studies, and thereby enhancing the accuracy of causal inference (Larsson et al., 2023). Drug target Mendelian randomization studies represent a straightforward application of this method. In these studies, genetic variants encoding protein targets have the potential to affect the expression of target genes. Furthermore, certain drugs can act on these targets, thereby influencing gene expression (Schmidt et al., 2020). In this study, we apply MR analysis to investigate the causal relationship between blood lipids and LE. We also examine the impact of lipid-lowering drugs on LE, aiming to discover new medications for the treatment of this condition.

## 2 Materials and methods

**FIGURE 1:**
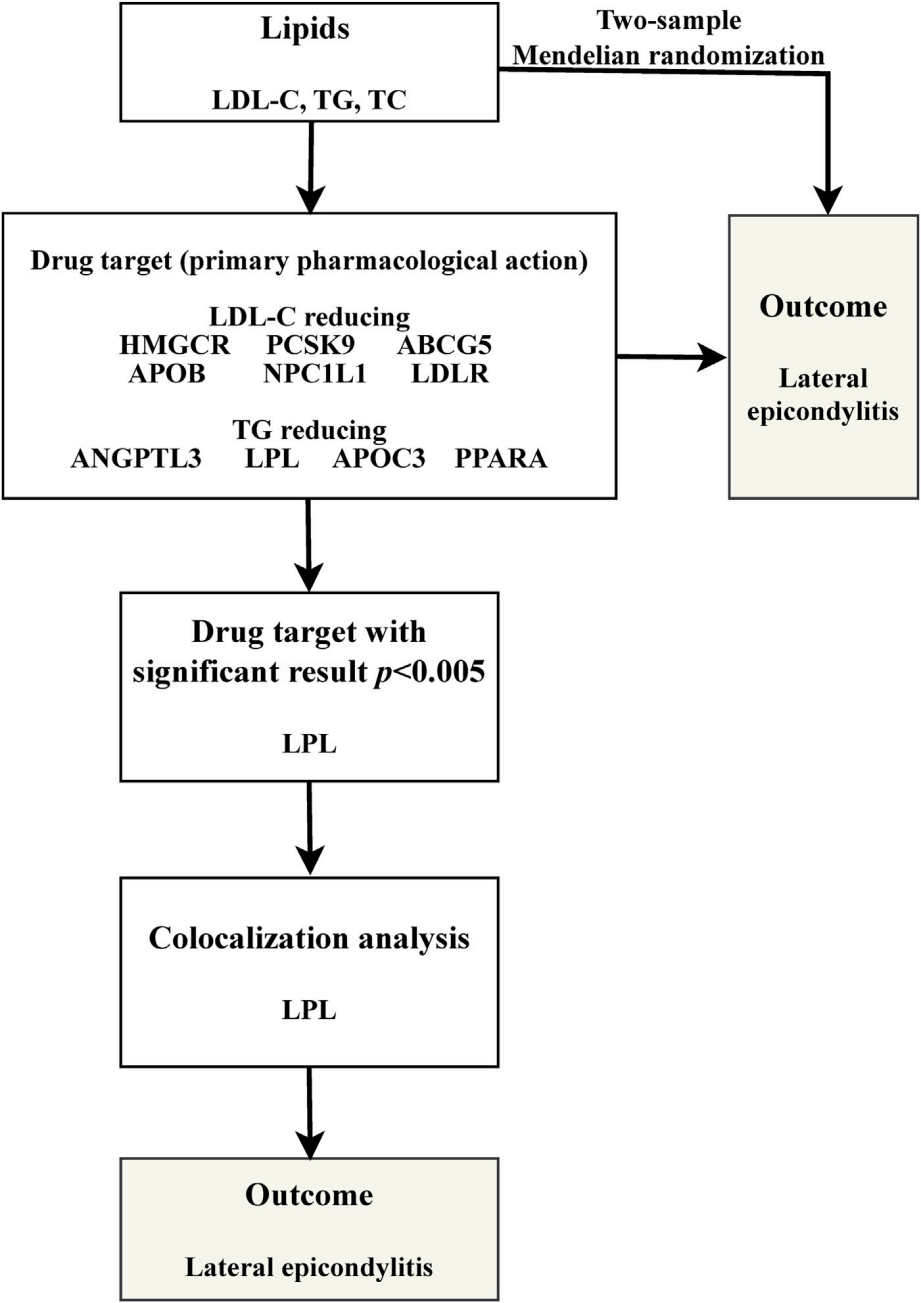
Flowchart of full-text.

**FIGURE 2:**
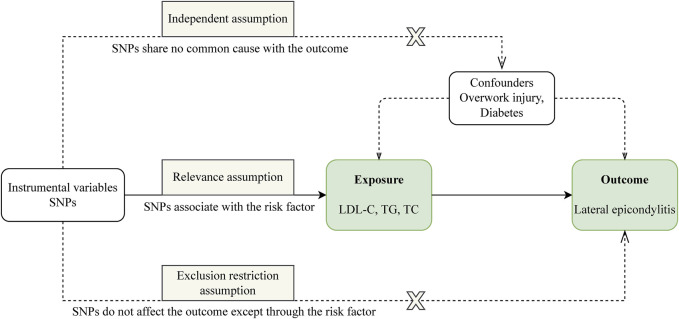
Three assumptions of Mendelian randomization.

Our study is structured as follows: 1) We perform a two-sample MR analysis to investigate the potential causal relationship between three lipid traits and LE; 2) We undertake a drug-target MR analysis involving 10 genes associated with blood lipids and LE, aiming to explore the potential link between these target genes and the condition; 3) For genes identified as significant in the drug-target analysis, we proceed with further colocalization analysis. Our comprehensive research approach is illustrated in Figure 1. The foundation of MR lies in three critical assumptions. Firstly, Relevance: The genetic variants chosen must be strongly associated with the exposure factor, serving effectively as proxies for exposure. Secondly, Independence: These genetic variations should not be linked to other confounding factors that may influence the outcome, thus ensuring the accuracy of the results. Lastly, Exclusion Restriction: The influence of genetic variations on the outcome should occur exclusively through the exposure factor, eliminating any other direct influence pathways. These essential principles are detailed in Figure 2.

We obtained the genome-wide association study (GWAS) data for lipid traits from the IEU Open GWAS database (https://gwas.mrcieu.ac.uk/), which includes data from the UK Biobank and the Global Lipids Genetics Consortium (GLGC). The sample sizes for LDL-C, TG, and TC were 440,546, 441,016, and 187,365, respectively. For our analysis, instrumental variables were carefully chosen based on stringent criteria: a linkage disequilibrium (LD) clumping threshold of r^2^ < 0.001, a *p*-value less than 5 × 10^−8^, and a physical distance threshold not exceeding 10,000 kb. We then utilized the PhenoScanner database (http://www.phenoscanner.medschl.cam.ac.uk/) to identify and exclude SNPs that could be associated with potential confounding factors, such as overuse and diabetes (Ranger et al., 2016; Cannata et al., 2021; Park et al., 2021). Ultimately, for our two-sample MR analysis, we included 127, 223, and 74 SNPs for LDL-C, TG, and TC, respectively. Outcome data on LE were obtained from the FinnGen(R9), which included 4,278 patients and 275,212 healthy controls. Detailed information on the GWAS data is provided in Supplementary Table S1, and the SNPs for each of the three lipid traits are listed in Supplementary Tables S2–S4.

We retrieved lipid-lowering drugs and their associated coding genes from the DrugBank database (https://go.drugbank.com/). From this database, we identified 10 relevant genes, including six genes associated with lowering LDL-C and four genes involved in reducing TG levels. Our methodology for selecting genetic variants was in line with established research practices. Within a 100 kb range surrounding these target genes, we identified single-nucleotide polymorphisms (SNPs) linked to LDL-C, TG, and TC levels. These SNPs demonstrated genome-wide significant associations in a GWAS meta-analysis conducted by the GLGC (*p* < 5 × 10^−8^). To further refine our selection, we clumped these SNPs at a linkage disequilibrium (LD) threshold of r^2^ < 0.20 and a physical distance threshold of 250 kb, effectively choosing them as proxies for the targets of lipid-lowering drugs. Following these steps, we conducted drug-target MR studies, adhering to similar principles. We selected coronary heart disease as a positive control in these studies to validate the effectiveness of the genetic instrumental variables we identified.

Colocalization analysis was a key component in verifying the validity of instrumental variable assumptions. This analysis was crucial for confirming that the observed associations between exposure and outcome were not confounded by different genetic variants in linkage disequilibrium. Out of the five hypotheses generated by this method, two warrant special attention: H3, which posits that the two traits, although correlated, are influenced by distinct causal variations; and H4, which suggests that these traits are not only correlated but also share a common causal variation. We estimated the colocalization probability by calculating the ratio H4/(H3 + H4). A ratio exceeding 80% was interpreted as a positive result (Zuber et al., 2022; Bi et al., 2023).

### 2.1 Statistical analysis

In our MR study, we utilize five methods to calculate associations. The core methods include Inverse Variance Weighted (IVW), MR Egger, and Weighted Median, which serve as the primary reference methods for our analysis. We assess heterogeneity using Weighted Cochran’s Q and MR Egger. Additionally, the egger-intercept parameter is employed to detect the presence of pleiotropy. The MR-PRESSO method plays a crucial role in identifying outliers, and it allows us to provide a causal estimate after excluding these outliers (Verbanck et al., 2018). To ensure statistical rigor, we apply the Bonferroni correction to adjust significance levels. For the analysis of three lipid traits, a *p*-value of less than 0.016 (0.05/3) is considered statistically significant. In the case of the ten lipid target genes, a *p*-value of less than 0.005 (0.05/10) is indicative of statistical significance. “Two-Sample MR,” and “coloc” in R (version 4.2.2) were used for all statistical analyses.

## 3 Results

### 3.1 The relationship between lipid traits and LE

**FIGURE 3:**
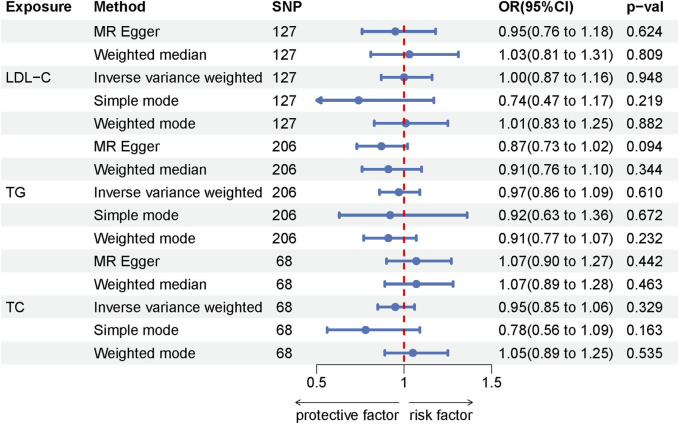
Forest plot of the MR results for the lipids-LE.

Among the lipid traits studied, LDL-C, TG, and TC were associated with 127, 223, and 74 independent SNPs, respectively, all exhibiting F-statistics values greater than 10 (ranging from 26 to 3,279), thus confirming their statistical validity. In our MR study investigating the relationship between these three lipid traits and LE, all applied analytical methods yielded *p*-values greater than 0.05. This suggests no evidence of a causal association between the lipid traits and LE. Moreover, the absence of heterogeneity as indicated by the Cochran Q test and the MR-Egger heterogeneity test, along with the MR-Egger intercept not showing signs of horizontal pleiotropy, further substantiates the robustness of our findings. The results of the MR analysis are detailed in Supplementary Table S5, while the assessments for heterogeneity and pleiotropy are documented in Supplementary Table S6. Additionally, the Forest plot illustrating the MR results is presented in Figure 3.

### 3.2 The relationship between lipid-lowering drug targets and LE

**FIGURE 4:**
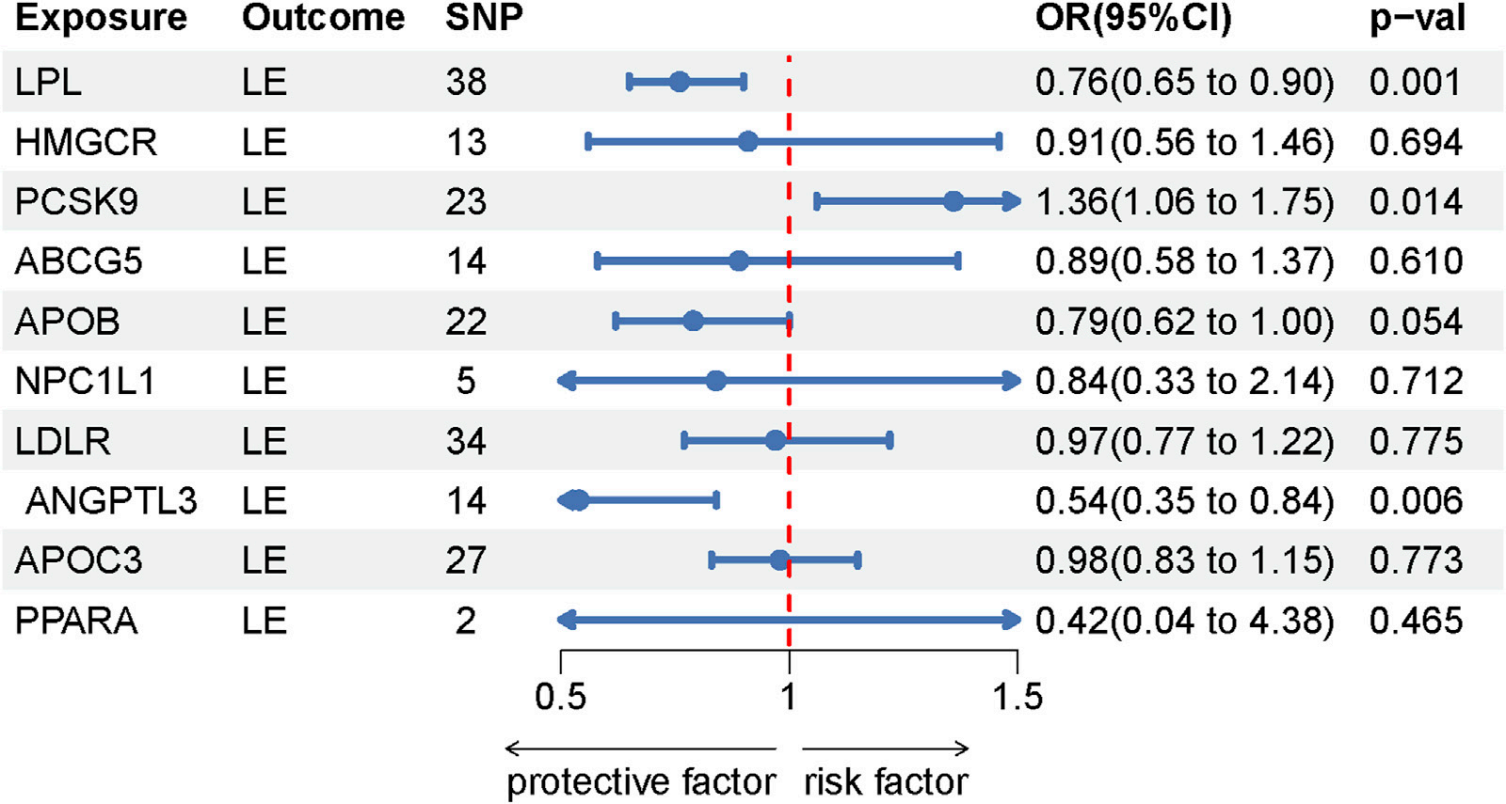
Forest plot of the drug-targeted MR using the IVW method.

**FIGURE 5:**
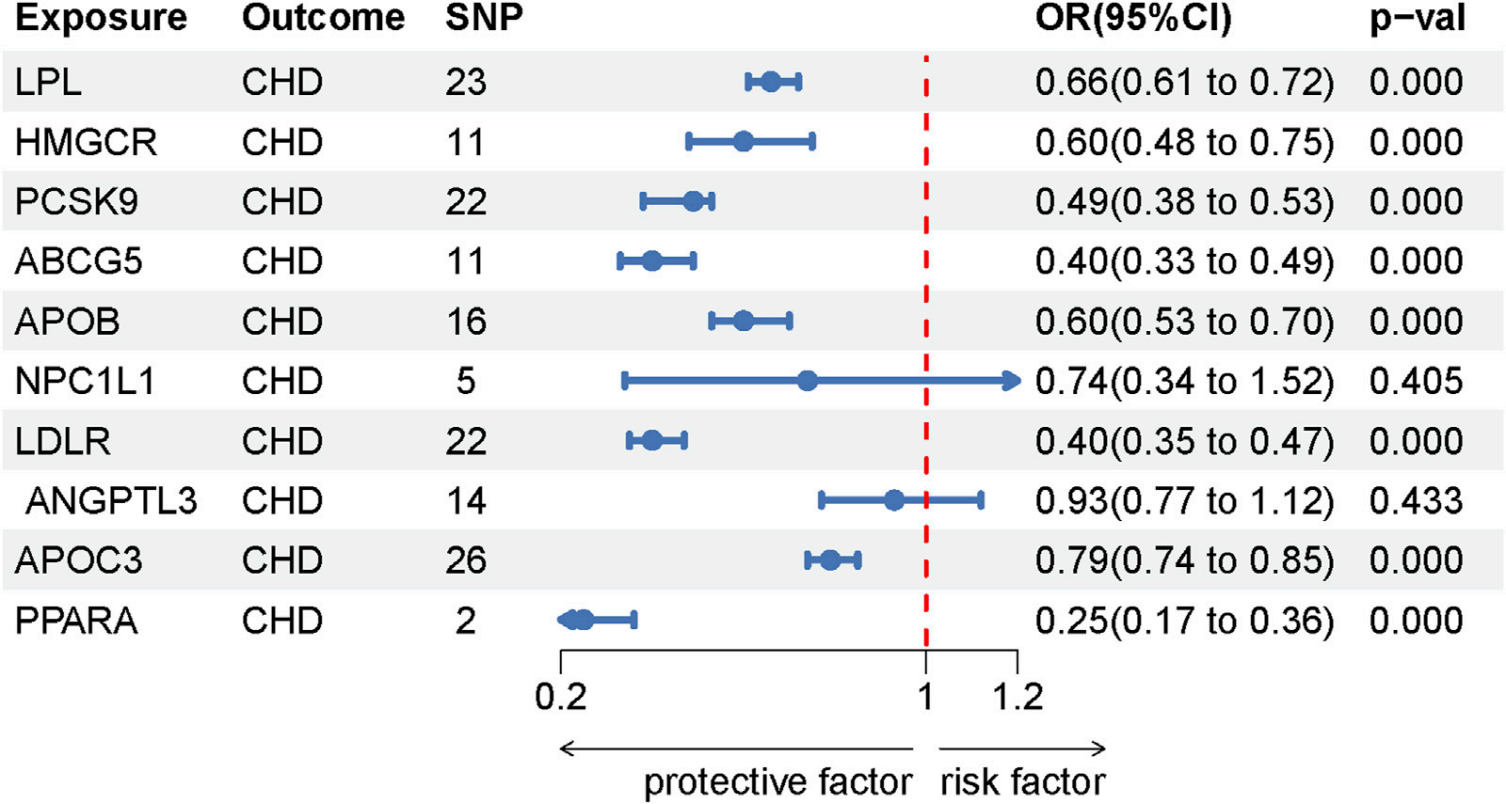
Forest plot of the MR results for the positive control using the IVW method.

**FIGURE 6:**
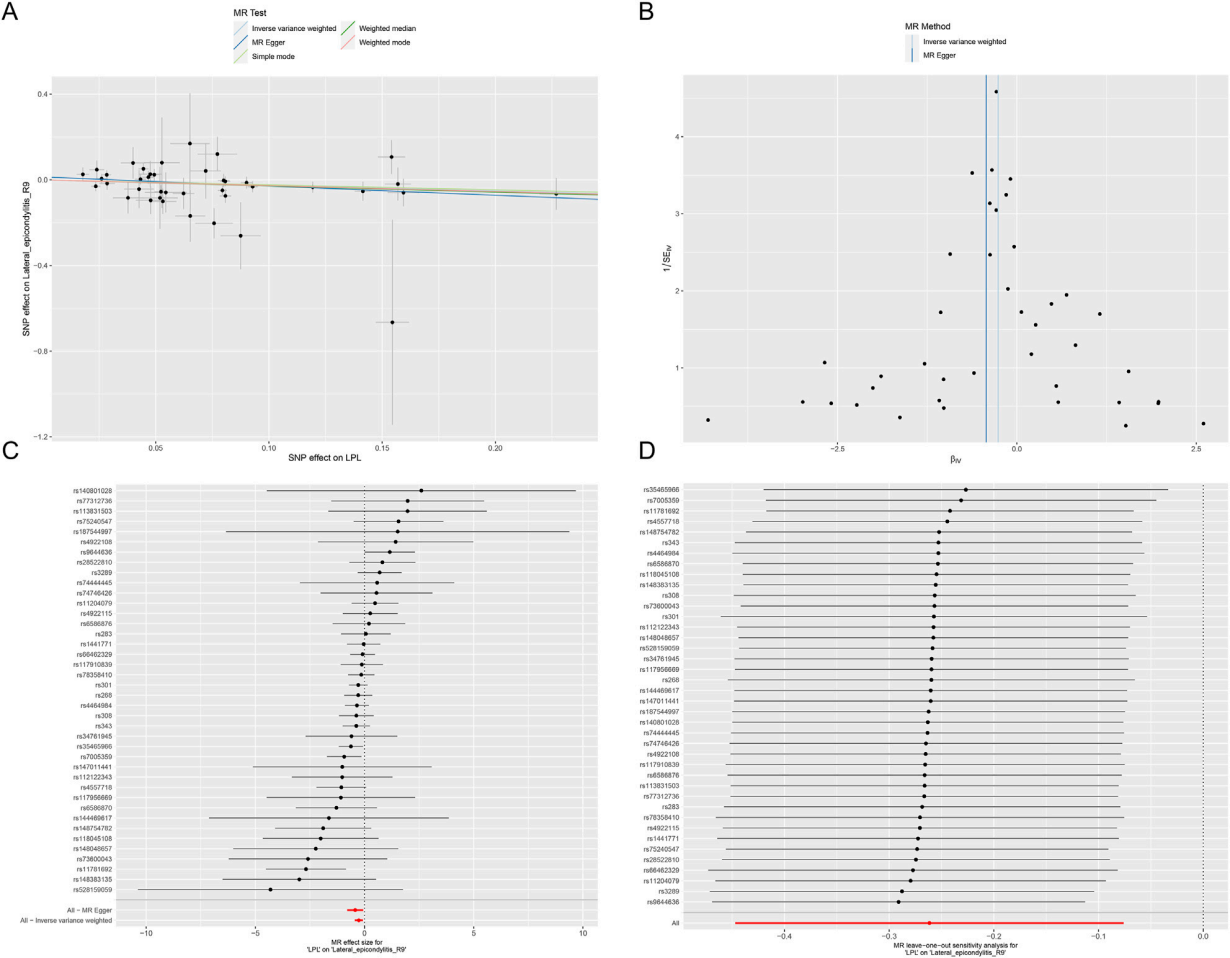
The visual graph of the drug target MR results for LPL-LE. **(A)** Scatter plot. **(B)** Funnel plot. **(C)** Forest plot. **(D)** Leave-one-out sensitivity analysis.

This study identified ten target genes—HMGCR, PCSK9, ABCG5, APOB, NPC1L1, LDLR, ANGPTL3, LPL, APOC3, and PPARA—associated with LE. Post-screening, all SNPs related to these genes demonstrated F-values greater than 10, ranging from 34 to 138,055, ensuring robust statistical significance. We employed coronary heart disease (CHD) as a positive control in our MR analysis. The results indicated that eight of the drug targets were significantly correlated with a decreased risk of CHD, thereby validating the instrumental variables used in the study. While the associations between NPC1L1, ANGPTL3, and coronary heart disease did not reach statistical significance, an overall protective trend against CHD was observed. Notably, the MR-Egger heterogeneity test revealed heterogeneity in the NPC1L1 target (*p* = 0.02). In contrast, LPL showed a significant association with a reduced risk of LE (OR = 0.76, [95% CI, 0.65–0.90], *p* = 0.001). However, no significant associations were found between LE and the other nine drug targets. The Cochran Q test and MR-Egger heterogeneity test for all drug targets yielded *p*-values greater than 0.05, suggesting a lack of heterogeneity across the study. The MR-Egger intercept test identified pleiotropy in the PCSK9 target (*p* = 0.02), but PCSK9 was not deemed a positive drug target. Detailed data on the ten target genes are presented in Supplementary Tables S7, S8. Supplementary Tables S9, S10 contain the MR results for drug targets, including tests for heterogeneity and pleiotropy. Supplementary Tables S11, S12 display the MR results for the positive control, along with checks for heterogeneity and pleiotropy. The IVW method’s drug-targeted results are shown in Figure 4. Figure 5 illustrates the MR results for the positive control using the IVW method. Figure 6 shows the visual graph of the drug target MR results for LPL-LE. (A) Scatter plot. (B) Funnel plot. (C) Forest plot. (D) Leave-one-out sensitivity analysis.

### 3.3 Colocalization analysis

In the study of the LPL target, we extended our analysis to include colocalization. This analysis revealed that the likelihood of a shared causal relationship between LPL expression in the blood and LE stands at 12.07% (H4). Conversely, the probability of there being different causal variants was found to be 1.93% (H3). The overall probability of colocalization, calculated as 86.2% (H4/H3+H4), suggests a strong likelihood of shared causality. Detailed results of this colocalization analysis are presented in Supplementary Table S13.

## 4 Discussion

The findings of this MR study suggest that blood lipids are not causally linked to LE. Furthermore, LPL emerges as a promising drug target for treating LE. Intriguingly, the protective role of LPL in LE seems independent of its lipid-lowering properties, indicating a potential alternative mechanism of action.

Current research findings have not established LDL-C, TG, and TC as risk factors for LE, aligning with conclusions from earlier studies (Scott et al., 2015; Sayampanathan et al., 2020; Park et al., 2021). Furthermore, recent MR studies indicate that TG is not a risk factor for conditions such as rotator cuff syndrome and achilles tendinopathy (He et al., 2022; Cao et al., 2023). Given that LE is categorized as a type of tendinopathy, these findings imply a lack of causal link between TG and LE. Our MR study specifically addresses this research gap, providing valuable insights into the relationship between these factors and LE.

The LPL gene is located on human chromosome 8p22, encoding 448 amino acids and comprising 10 exons (spanning 30 kb) interspersed with nine introns (spanning 6 kb) (He et al., 2018). LPL is ubiquitously expressed in the capillary endothelium of all tissues, where its primary function is to hydrolyze TG into glycerol and free fatty acids (FFA), which are then absorbed by tissues (Geldenhuys et al., 2017b). The gene is most abundantly expressed and active in adipose tissue, skeletal muscle, and the heart, while its expression is comparatively lower in other tissues (Chang, 2019). LPL exhibits tissue-specific physiological roles: in skeletal muscle, it facilitates the breakdown of plasma lipoproteins, reducing plasma TG and providing FFA for oxidative energy production, whereas in adipose tissue, the FFA generated is preferentially directed toward lipid storage (Li et al., 2014). Environmental factors such as fasting/feeding, exercise, and cold exposure can modulate LPL protein levels and activity, thereby channeling fatty acids to specific cells according to their energy requirements (Wu et al., 2021). Furthermore, LPL expression is tightly regulated at both the transcriptional and translational levels through multiple complex mechanisms (Kersten, 2014; Geldenhuys et al., 2017b).

In our study, we discovered that LPL is linked to a lower risk of LE. This contrasts with ANGPTL3 and APOC3, which, despite also lowering TG, do not show a similar association. This leads us to hypothesize that the protective effect of LPL against LE might not be related to its lipid-lowering properties but rather to its role in reducing inflammation. Recent research over the past decade has emphasized the significant involvement of inflammatory cells and mediators in the progression of tendinopathy (Dakin et al., 2014; Millar et al., 2017). The literature describes the initial phase of LE as an acute inflammatory response, which precedes changes in tissue structure, such as angiogenesis and disordered collagen fiber alignment (Ackermann and Renström, 2012; Ahmad et al., 2013). Confirming this, a color Doppler ultrasound study on LE identified the presence of inflammation (Torp-Pedersen et al., 2008). Cells associated with inflammation, including macrophages, mast cells, B lymphocytes, and T lymphocytes, heavily infiltrate the affected area, releasing inflammatory factors like Interleukin-1 (IL-1), Interleukin-6 (IL-6), Tumor Necrosis Factor-alpha (TNF-α), and Tumor Necrosis Factor-beta (TNF-β). This leads to increased activity in the Nuclear Factor-kappa B (NF-κB) pathway, triggering an inflammatory cascade (Han et al., 2012; Millar et al., 2021). Beyond its triglyceride-lowering function, LPL has been reported to suppress the release of inflammatory factors such as TNF-α, IL-6, and Cyclooxygenase-2 (COX-2) by inhibiting NF-κB activation, thereby mitigating inflammation (Kota et al., 2005; Takasu et al., 2012). This inhibition of inflammation may be a key mechanism through which LPL treats tendinopathies.

Discovered in the early 1990s, NO – 1886 (Ibrolipim) is an established LPL activator. It effectively increases LPL mRNA levels in tissues, boosts the concentration of LPL protein in plasma following heparin administration, and enhances LPL activity. In addition, NO – 1886 has been shown to lower plasma triglyceride levels in animals with lipid disorders (Yin et al., 2004; Geldenhuys et al., 2017a). Animal studies further reveal that this compound significantly reduces 29 types of cellular inflammatory factors, including IL-1, IL-6, and TNF-β, among others. It also suppresses the expression of most pro-inflammatory cytokines in adipose tissue, leading to reduced tissue inflammation (Cai et al., 2006). Evidence suggests that NO – 1886 primarily targets LPL in skeletal muscle (Kusunoki et al., 2005), indicating its potential to mitigate tendon inflammation. Our study findings also demonstrate that ANGPTL3 inhibitors display a protective trend against LE, with an odds ratio of 0.54 ([95% CI, 0.35–0.84], *p* = 0.006). Moreover, the literature indicates that ANGPTL inhibitors enhance LPL activity, urging further exploration of the link between ANGPTL3 and LE. The newly reported drug molecule C10d exhibits at least double the LPL activation efficiency of N0-1886 and can reverse the inhibitory effect of ANGPTL on LPL, increasing its investigative potential (Geldenhuys et al., 2014; 2017b). In conclusion, drugs that activate LPL may prevent LE by inhibiting the release of inflammatory factors. However, further cellular and molecular-level studies are needed to explain this result, and high-quality clinical research is also crucial.

It is important to note that drug-target MR results suggest an association between PCSK9 inhibition and an increased risk of LE (OR = 1.36, [95% CI, 1.06–1.75], *p* = 0.014), a finding that may appear counterintuitive. However, this result demonstrates pleiotropy (*p* = 0.02, Supplementary Table S10), implying the presence of confounding factors that invalidate the conclusion. The relationship between PCSK9 inhibition and inflammation remains a subject of debate. Seidah et al. reported that PCSK9 inhibition suppresses TLR4/NF-κB signaling, leading to the inhibition of inflammatory mediators (Seidah and Prat, 2021). Similarly, Ding et al. found that PCSK9 inhibition reduces the inflammatory response by suppressing toll-like receptors (TLRs), the NLRP3 inflammasome, and the NF-κB pathway (Ding et al., 2022). Conversely, Pradhan et al. observed that in a cohort of 9,738 patients treated with PCSK9 inhibitors for 14 weeks, 47.2% exhibited residual inflammatory risk, with high-sensitivity C-reactive protein (hs-CRP) levels ≥2 mg/L during treatment (Pradhan et al., 2018). Given the inconclusive relationship between PCSK9 inhibition and inflammation, coupled with the lack of a positive drug target MR result in this study, further research is needed to clarify the connection between PCSK9 inhibition and LE.

This study possesses several limitations. Primarily, our reliance on data from FinnGen as the exclusive outcome may lead to certain biases. Additionally, the absence of RCTs focusing on the risk factors for lateral epicondylitis limits the breadth of our analysis. In excluding confounding SNPs, we specifically targeted those associated with overuse and diabetes, which could introduce bias stemming from subjective selection. Finally, as our study population was predominantly European, it is important to consider that the findings might not be universally applicable across different ethnic groups.

## 5 Conclusion

Low-density lipoprotein cholesterol, triglycerides, and total cholesterol are not risk factors for lateral epicondylitis. Moreover, we identified lipoprotein lipase (LPL) as a potential therapeutic target, suggesting its importance in preventing and treating this condition.

## Data Availability

The original contributions presented in the study are included in the article/Supplementary Material, further inquiries can be directed to the corresponding author.
